# Association of the presence and its types of lamina fractures with posterior dural tear and neurological deficits in traumatic thoracic and lumbar burst fractures

**DOI:** 10.1186/s12891-021-04178-9

**Published:** 2021-03-23

**Authors:** Xuchao Shi, Shate Xiang, Bo Dai, Zhennian He

**Affiliations:** 1 Department of Orthopedics Surgery of Beilun People’s Hospital, No. 1288, Lushan East Road, Ningbo, 315800 Zhejiang Province China; 2 College of Medical Technology, Zhejiang Chinese Medical University, No. 548, Binwen Road, Binjiang District, Hangzhou, 310053 Zhejiang Province China

**Keywords:** Vertical laminar fractures, Coronal plane, Dural tears, Treatment, Spinal lesions

## Abstract

**Introduction:**

The appropriate and optimal treatment for thoracic and lumbar (TL) burst fractures remains a topic of debate. Characterization of vertical laminar fractures (coronal cross-sectional imaging) is presented in this study to determine the severity and treatment options in TL burst fractures.

**Methods:**

A retrospective evaluation of 341 consecutive patients with TL burst fractures was divided into Group I (whole), Group II (partial), and Group III (intact) based on the vertical laminar fracture morphology from coronal images on computed tomography (CT) scans. The presence of preoperative neurological status was reviewed, and several radiological parameters were measured. In addition, the incidence of dural tears was calculated in patients that underwent a decompression with posterior approach.

**Results:**

In total, 270 lumbar and 71 thoracic burst fractures were analyzed. Compared with the intact group, the two other groups had significantly shorter central canal distance, wider interpedicular distance, and smaller spinal canal area, in particular, Group III. The incidences of preoperative neurological deficits in Groups I to III were 63.0, 22.2, and 6.3%, respectively. The incidences of dural tears in Groups I to III were 25.6, 6.3, and 0%, respectively.

**Conclusion:**

The morphology of vertical laminar fractures observed across the coronal plane was important. Patients with “whole”, “partial” and “intact” laminar fractures indicated different severity of TL burst fractures. Due to the high probability of dural tears, decompression is recommended as a primary intervention for patients with “whole” laminar fractures. However, for patients without vertical laminar fractures, minimally invasive technique might be a better choice to avoid approach-related complications.

## Introduction

Thoracic and lumbar (TL) burst fractures have been reported for several decades [1]. Under axial loading during trauma, the vertebral body collapses and the interpedicular distance increases. Because of the connections between the pedicles and laminae, TL burst fractures are usually associated with laminar fractures [2].

The correlation between the presence of a laminar fracture and a dural tear as well as between the presence of a dural tear and a severe neurological deficit have been well documented [3–6]. Studies into the morphology [3–5, 7], the incidence of dural tears, nerve root entrapment, and spinal cord compression on axial cross sectional anatomy were quite a lot [4, 5, 7]. However, the importance to the coronal morphology of laminar fractures appears to be underestimated for its indispensability to assess stability across the fracture segment.

This study aims to determine the characteristic fracture morphology, preoperative neurological status, and radiological findings of patients with vertical laminar fractures. In addition, the significance of the coronal morphology of laminar fractures is emphasized when choosing the appropriate treatment for TL burst fractures.

## Methods

A total of 341 consecutive patients with acute (within a 10 day history) one level spinal burst fractures [2] were included in this study. Patients were diagnosed and treated in hospital from 2012 to 2018. Exclusion criteria included pathologic fractures, multiple segmental injuries, patients with chronic, or age-indeterminate thoracic (or both) and lumbar trauma (or both), inability to undergo radiological evaluation, and those with CT datasets from only one imaging plane. The preoperative neurological status was reviewed from the medical charts and operative notes. A proportion of patients were explored by decompression with posterior approach; then, if there were any dural tears and entrapped nerve roots, they were repaired [8, 9]. Following decompression, the short segment transpedicular screw fixation techniques and fusion were performed.

**Fig. 1:**
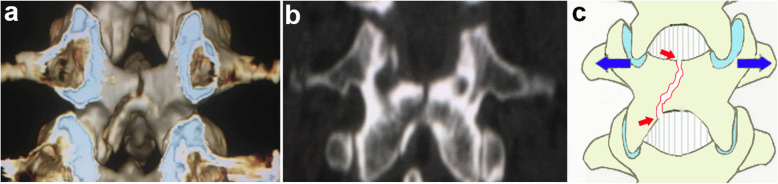
Reconstructed three-dimensional CT scan showed the whole laminar fractures directly (**a**). The coronal CT scan of whole laminar fractures (**b**). Illustration (**c**)

**Fig. 2:**
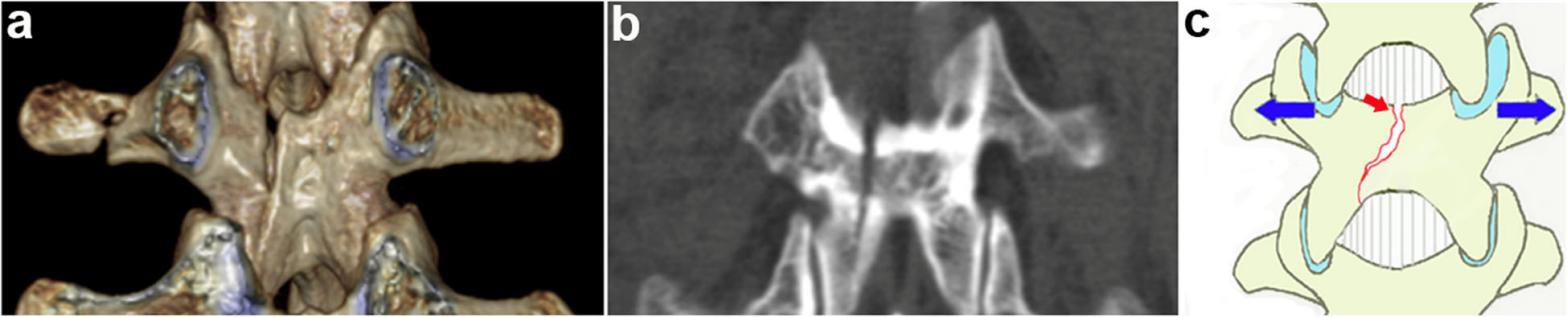
Reconstructed three-dimensional CT scan showed the partial laminar fractures directly (**a**). The coronal CT scan of partial laminar fractures (**b**). Illustration (**c**)

**Fig. 3:**
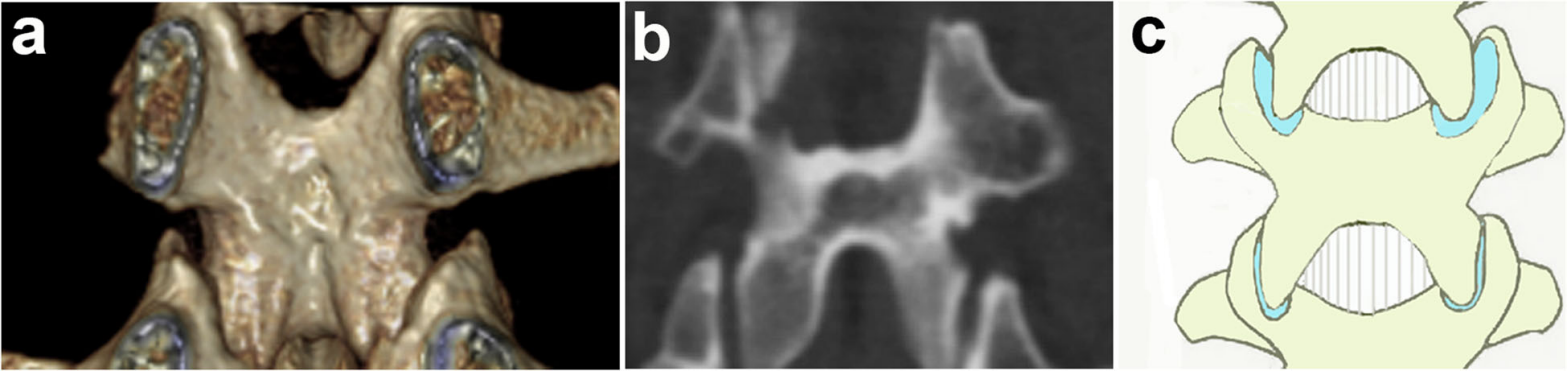
Reconstructed three-dimensional CT scan showing no laminar fracture (**a**). The coronal CT scan of no laminar fractures (**b**). Illustration (**c**)

Groups used to classify the laminar fracture pattern were: Group I = “whole: whole laminar fracture (with fractures in both the top and bottom of the lamina, Fig. 1, red arrows)”; Group II = “partial: partial laminar fracture (with fractures only in the top of the lamina, Fig. 2, red arrow)”; and Group III = “intact: without laminar fractures, Fig. 3”.

The percentages for the widening of the pedicles, compression of anterior vertebral height, narrowing of the central canal distance, and decrease of the spinal canal area in the fractured vertebra were established by comparing the same parameters with those of the vertebrae immediately above and below. The radiological parameters were measured using the Picture Archiving and Communication System (PACS; INFINITT PACS; INFINITT, Seoul, Korea) in CT scans. Statistical analysis was performed using IBM SPSS Statistics Version 22 (IBM-SPSS, Inc., Chicago, IL, USA). The analysis of variance was used for continuous variables and the Chi-squared test (χ^2^) or Fisher’s exact test (where appropriate) were used for categorical variables in overall difference statistical analysis. The *least significant difference t test* (LSD-t) was used for continuous variables and the χ^2^ partition was used for categorical variables in multiple comparisons. Differences were considered statistically significant at *P* < 0.05 and the differences were corrected as *P* < 0.0125 in sections of the χ^2^ method.

## Results

A total of 213 patients were male and 128 patients were female with an average age of 45.9 ± 12.6 years. They commonly presented with burst fractures of the TL vertebrae following falls from heights (62.46%) followed by simple falls (20.82%), and motor vehicle accidents (9.68%). A total of 71 patients had thoracic fractures and 270 patients had lumbar fractures.

Group I had 146 patients, Group II had 36 patients, and Group III had 159 patients. The analysis of variance demonstrated that there were significant differences in all the radiological parameters, except for the ratio of anterior height (*P* = 0.011), the rest were *P* < 0.001. In multiple comparisons, the difference among these groups was of great significance based on the ratio of central canal distance, interpedicular distance, and spinal canal area. For the ratio of anterior height, there were no significant differences between groups I and II and between groups I and III. However, the difference between groups I and III was significant (Table 1). The mean and standard deviations for the radiological parameters were given in Table 2.

**Table 1:** Multiple comparisons of variables from different laminar fracture groups (*P* value)

Variables	Group I	Group II	Group III
Ratio of central canal distance(%)			
Group I	–	–	–
Group II	0.001	–	–
Group III	*	*	–
Ratio of interpedicular distance(%)			
Group I	–	–	–
Group II	*	–	–
Group III	*	*	–
Ratio of spinal canal area(%)			
Group I	–	–	–
Group II	0.020	–	–
Group III	*	0.002	–
Ratio of anterior height(%)			
Group I	–	–	–
Group II	0.684	–	–
Group III	0.003	0.153	–

The asterisk (*) denotes *p* < 0.001

**Table 2:** Mean and standard deviation for radiological parameters for all groups

Variables	Group I	Group II	Group III
Ratio of central canal distance(%)	51.2 ± 20.5	62.4 ± 17.1	76.5 ± 15.7
Ratio of interpedicular distance(%)	123.1 ± 10.7	113.2 ± 7.8	104.2 ± 6.7
Ratio of spinal canal area(%)	59.3 ± 23.6	69.0 ± 23.0	81.6 ± 21.0
Ratio of anterior height (%)	66.0 ± 12.5	66.9 ± 10.6	70.0 ± 11.1

As given in Table 3, the demographic distributions in the presence of preoperative neurological deficits among groups I to III were 92 patients (63%), 8 patients (22.2%), and 10 patients (6.3%), respectively. The difference for this presentation was significant for all groups (*P* < 0.001). Based on multiple comparisons, the incidences of preoperative neurological deficits in the different groups were significantly different from each other (*P* < 0.0125).

**Table 3:** Distribution of frequencies of morphology of vertical laminar fractures in coronal plane according to the intact or deficits of preoperative neurological status

Morphology	Preoperative neurological status				Statistic test
	Intact		Deficits		
	N	Percentage	N	Percentage	
Overall difference statistical analysis					χ2 test
Group I	54	37.0%	92	63.0%	*P* < 0.001
Group II	28	77.8%	8	22.2%	
Group III	149	93.7%	10	6.3%	
Multiple comparisons					χ2 partition test
Group I	54	37.0%	92	63.0%	*P* < 0.001
Group II	28	77.8%	8	22.2%	
Group I	54	37.0%	92	63.0%	*P* < 0.001
Group III	149	93.7%	10	6.3%	
Group II	28	77.8%	8	22.2%	*P* = 0.007*
Group III	149	93.7%	10	6.3%	

The asterisk (*) denotes Fisher’s exact test

In the current study, 105 out of 341 patients underwent decompression with the posterior approach. In groups I to III 20 patients (25.6%), 1 patient (6.3%), and no patients (0%) with dural tears, respectively. A total of 58 patients (74.4%), 15 patients (93.8%), and 11 patients (100%) did not have dural tears, respectively.

In Table 4, Fisher’s exact test demonstrated a significant difference in patients with dural tears among these groups (*P* < 0.001). The χ2 partition test demonstrated that there was no correlation between groups I and II (*P* = 0.081), between groups II and III (*P* = 0.185) with dural tears. However, there was a significant difference between groups I and III (*P* < 0.001).

**Table 4:** Distribution of frequencies of morphology of laminar fractures in coronal plane according to the absence or presence of posterior dural tears

Morphology	Posterior dural tears				Statistic test
	Absence		Presence		
	N	Percentage	N	Percentage	
Overall difference statistical analysis					Fisher’s exact test
Group I	58	74.4%	20	25.6%	*P* < 0.001
Group II	15	93.8%	1	6.3%	
Group III	11	100%	0	0%	
Multiple comparisons					χ2 partition test
Group I	58	74.4%	20	25.6%	*P* = 0.081*
Group II	15	93.8%	1	6.3%	
Group I	58	74.4%	20	25.6%	*P* < 0.001
Group III	11	100%	0	0%	
Group II	15	93.8%	1	6.3%	*P* = 0.185*
Group III	11	100%	0	0%	

The asterisk (*) denotes Fisher’s exact test

## Discussion

The laminar fracture was a reliable factor to assess the severity of spinal lesions [1, 3], which usually occurred in TL burst fractures [2]. Because of axial loading, the vertebral body fractured into several fragments, both the pedicles and the posterior elements splayed laterally, which led to vertical laminar fractures. The more severe the injured vertebral body was the more damage the pedicles delivered to the lamina, which caused a difference in the morphology of vertical laminar fractures.

It was important to assess the severity (stable or unstable) of spinal fractures for treatment options and avoid neurologic deficits [1, 3–5, 7, 8, 10]. Laminar fractures in patients with burst spinal fractures were more severe and were associated with potential instability [1–3]. Therefore, the evaluation of morphology in TL burst fractures was important [11]. The characteristics of these injuries were demonstrated well by CT. It enabled the accurate measurement of the widening pedicles, compression of anterior vertebral height, narrowing of the central canal distance, and decrease of the spinal canal area in the fractured vertebra, and therefore, determination of the correlation between the severity of the lesion and neurological deficits.

Denis et al. [8] noted a vertical lamina fracture that occurred secondarily to the splaying of the posterior arch of the vertebra under axial loading and described these as a greenstick fracture of the anterior cortex of the lamina. However, previous studies only paid attention to axial CT scans and divided them into complete and incomplete types (greenstick) [3, 4, 7]. The axial morphology of laminar fracture [3–5, 7] and its associations with the severity of spinal lesions have been well documented in the literature [1, 3, 7]. In addition, researchers have discussed the incidence of dural tears [4, 5, 7] and have attempted to determine the specific clinical and radiological factors or intraoperative pathologic findings that predict dural tears and nerve root entrapment [4, 5]. Futhermore, Denis et al. [8] established the diagnosis and treatment of cauda equina entrapment in the vertical lamina fractures of lumbar burst fractures. However, it is noteworthy that the lamina is a three-dimensional structure and laminar fractures expand in the transverse plane and splay into the coronal plane. This is because the posterior wall of the spinal canal, the laminae are in contact with the dura sac directly. Compared with the axial morphology of laminar fractures, the coronal plane is of equal importance.

In this study, these groups showed significant differences in all the radiological parameters. Except for in anterior height, there were significant differences between Groups I and III in the remaining radiological parameters. Based on the results listed in Tables 1 and 2, patients with whole vertical laminar fracture were the severest group. These characteristic results reflected that the longer the vertical laminar fracture had existed, the more severe the injured vertebra was.

The preoperative neurological status among these groups was of significant difference. Patients with whole laminar fractures were more probable to be associated with a preoperative neurological deficit than those in the rest groups. In this study, 21 patients had dural tears. The reasons for the presence of posterior dural laceration following vertical laminar fractures might be as follows: (1) the posteriorly displaced dural sac was impaled on the sharp edges of the laminar fracture at the moment of trauma [6]; and (2) as the axial load dissipated, the laminar fracture fragments recoiled to restoration, possibly entrapping the dural sac and nerve roots [3], which led to a neurologic deterioration [3, 4]. Because of much longer laminar fractures, patients in Group I contained fractures with more sharp edges that had more contact surface with the laminar fractures, which led to the dural tears occurring more easily. This assumption was reached combined with the results detected in patients that were explored with a posterior approach in our study. The incidences of dural tears in Groups I to III were 25.6, 6.3, and 0%, respectively.

In TL burst fractures, for the treatment of cases with neurological injuries, internal fixation following decompression is widely accepted [12]. However, surgical decompression is controversial in patients who are neurologically intact [13]. In lumbar burst fractures, it has been suggested that patients associated with greenstick laminar fractures [4, 7], or radiological evidence of posterior displacement of the neural elements in injured vertebrae [8] require posterior surgical exploration. However, dural tears might cause the diffusion of blood within the subdural space, cerebrospinal fluid leaks, pseudomeningocele formation, entrapment of herniated nerve roots, and the delayed scarring of neural structures [5, 14–17]. Therefore, in Group I, due to the high probability of and severe complications due to dural tears, when whole laminar fractures occur, it is recommended that posterior exploration is used to expose the dura safely before any reduction maneuver. However, for Group III, which do not have dural tears and have a low incidence of preoperative neurological deficits, patients that received minimally percutaneous methods for spinal fixation avoided approach-related complications [18]. Minimally invasive techniques, as shown in the 2-year follow-up study, gave at least the same results as those achieved using the open technique in clinical, functional, and radiological results. However, the blood loss, postoperative pain, and surgical time were significantly reduced using minimally invasive techniques [19]. A systematic review and meta-analysis of comparative studies [20] were consistent with these findings.

As with any database study, the limitations of the conclusions in our study must be noted. First, the demographic distributions in each group were various. Both Groups I and III had nearly 45% patients and there were only 36 individuals in Group II. In addition, due to a relative low incidence of such cases described in this study, multicenter studies with larger sample size will be necessary. Anyhow, for the first time, we described the morphology of vertical laminar fractures in TL burst fractures in coronal plane, revealed its association with the severity of spinal injury, and mentioned the significance in choosing the appropriate treatment.

## Conclusions

Collectively, our study first demonstrates the association of the presence and its types of lamina fractures with posterior dural tear and neurological deficits in traumatic thoracic and lumbar burst fractures.

## Data Availability

The datasets used and/or analyzed during the current study are available from the corresponding author on reasonable request.

## Abbreviations

TL: Thoracic and lumbar
CT: Computed tomography
PACS: Picture Archiving and Communication System
χ^2^: Chi-squared test
LSD-t: *least significant difference t test*
